# Precision and reproducibility of T_2_ quantifications in myocardial T_2_ mapping: impact of the number of echoes and reconstruction model

**DOI:** 10.1186/1532-429X-17-S1-W9

**Published:** 2015-02-03

**Authors:** Tamer Basha, Mehmet Akcakaya, Sébastien Roujol, Reza Nezafat

**Affiliations:** 1 Cardiology, BIDMC, Boston, MA, USA; 2 Harvard Medical School, Boston, MA, USA

## Background

Quantitative myocardial T_2_ is a promising technique to assess myocardial inflammation and edema (1). Recent implementations have utilized T_2_-prepared (T_2_prep) SSFP sequences to acquire a multiple T_2_ weighted images at different echo times, and generate T2 maps based on a 2-parameter (2P-fit) model of T_2_ decay (2,3). Recently, a 3-parameter fitting (3P-fit) model was found superior to the conventional 2P-fit model, as it compensates for T_1_ relaxation effect, and results in more accurate T_2_ measurements (4). In this work, we sought to characterize the 3P-fit approach in terms of precision and reproducibility and to evaluate the influence of the number of employed T_2_prep echo times on these two metrics.

## Methods

Monte-Carlo simulations (1000 repetitions) were performed to study the effect of increasing the number of T_2_prep images. Block equation was used to simulate the signal intensities of a presumed tissue of T_2_ = 50ms at different T_2_prep echo times and different SNR levels. T_2_ was then estimated using a 2- and 3-parameter fitting model, and the precision was quantified for each model. Ten healthy subjects (27±10 y/o, 5m) were then imaged using a 1.5 T Phillips scanner with a free-breathing ECG-triggered single shot T_2_prep bSSFP sequence (FOV = 320×320 mm^2^, in-plane resolution = 2.5×2.5 mm^2^, slice thickness = 8mm, TR/TE = 2.2/1.1ms, FA = 40°, SENSE rate = 2, acquisition window = 140 ms, 14 T_2_prep echo times = 0,25,35,…135,145 ms). A 4s rest period after each image to allow for full spin relaxation. Data were reconstructed using the 3P-fit model. For comparison, a conventional T_2_ mapping sequence was acquired (Breath hold, 3 T_2_prep echo times = 20,50,75ms, and 2P-fit model). For each subject, both sequences were repeated 5 times. Precision and reproducibility were compared using different subset of T_2_prep echo times. Based on these results, an optimized T_2_ mapping sequence using 10 T_2_prep echoes and a 3P-fit model is proposed and evaluated in-vivo in 10 healthy subjects (29±17 y/o, 4m). This sequence is compared to the same conventional T_2_ mapping sequence in term of precision and reproducibility.

## Results

**Figure 1:**
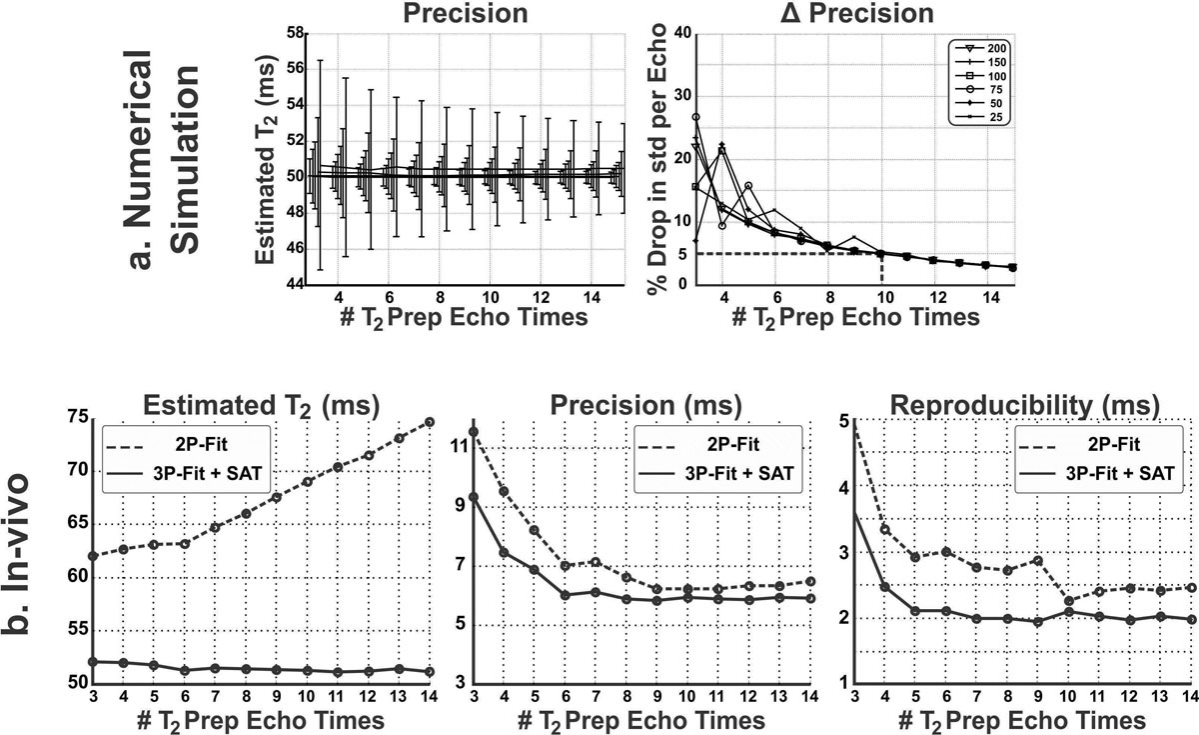
a) Numerical simulation results for the effect of number of echo images on the precision of the quantifications for different signal-to-noise ratios. As the number of echoes increases, the precision gets better till it nearly saturates for number of echoes ≥ 10. b) Accuracy, precision and reproducibility of T_2_ mapping when using different number of echo images. With increasing the number of echoes, estimated T_2_ values changes significantly when using 2-pt fits, while it shows consistency when using the 3-pt fits regardless of the number of echoes used for the estimation. Both precision and reproducibility increases when using more echo images for the T_2_ estimation. However, and similar to what numerical simulations predicts, the effect nearly saturates for number of echoes ≥ 10.

**Figure 2:**
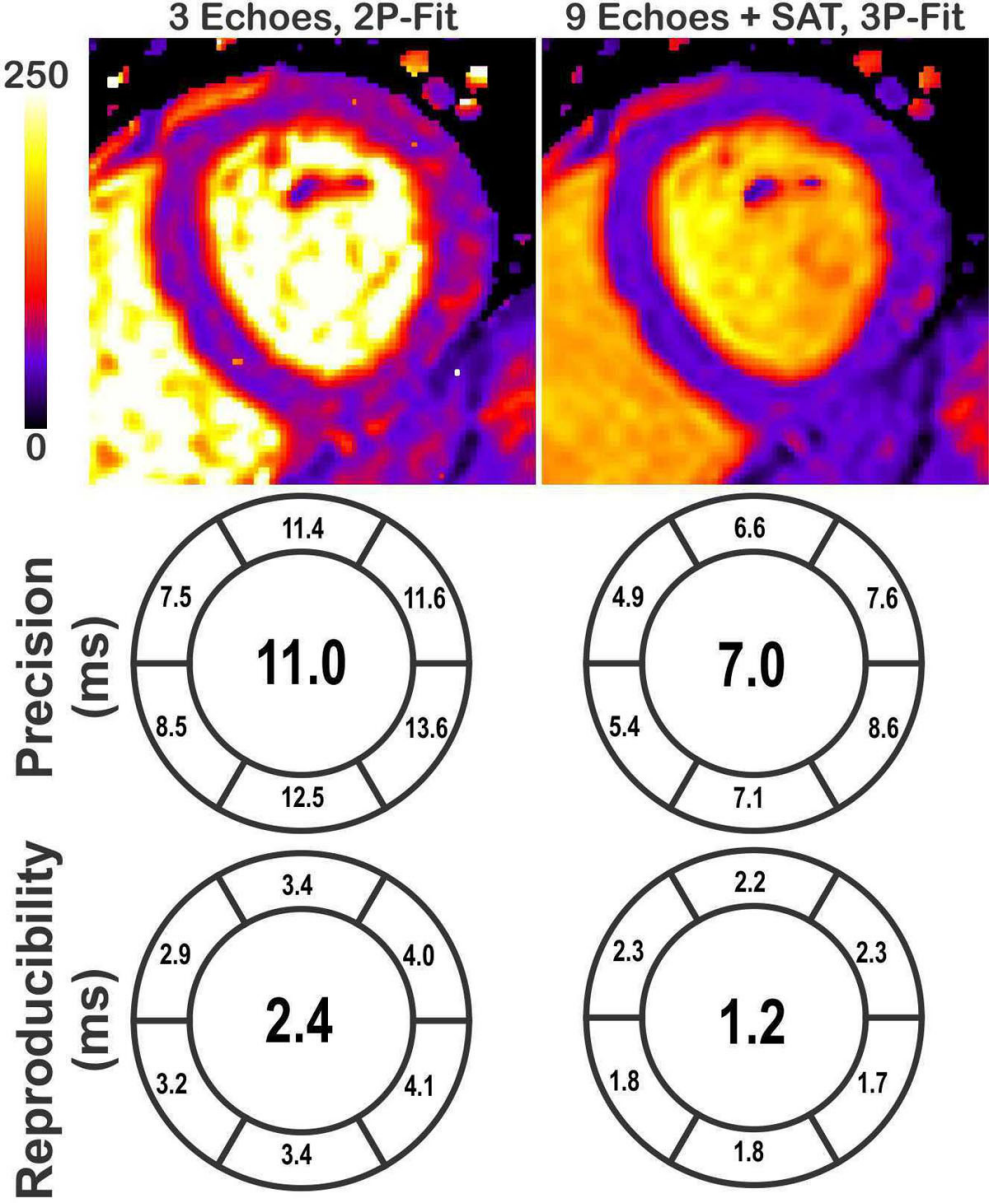
An example for the T_2_ maps of one healthy subject. The bull's-eyes shows the overall precision and reproducibility among the 10 subject in a segment-based analysis, when using the 3 echoes with 2-pt fit, and 10 echoes with 3-pt fit.

T_2_ measurements using a 2P-fit model are dependent on the number of T_2_prep echo times (Figure 1). The 3P-fit model provides T_2_ measurements independent from the number of T_2_prep echo times. Higher precision and reproducibility was achieved with increased number of T_2_prep echo times. Improved in-vivo precision and reproducibility was achieved using the proposed sequence when compared to the conventional sequence (7ms vs. 11ms p=XX and 1.2ms vs. 2.4ms p=XX, respectively) (Figure 2).

## Conclusions

The proposed sequence using 10 T_2_prep echo times and a 3P-fit model is independent from the number of T_2_prep echo times and provides better in-vivo precision and reproducibility than the conventional technique.

## Funding

N/A.
